# The genome sequence of the Rose Chafer, *Cetonia aurata *(Linnaeus, 1758)

**DOI:** 10.12688/wellcomeopenres.20412.2

**Published:** 2026-05-05

**Authors:** Andrew Grayson, Michael Geiser, Natural History Museum Genome Acquisition Lab, Darwin Tree of Life Barcoding collective, Wellcome Sanger Institute Tree of Life programme, Wellcome Sanger Institute Scientific Operations: Sequencing Operations collective, Tree of Life Core Informatics collective, Darwin Tree of Life Consortium

**Affiliations:** 1 Independent researcher, York, England, UK; 2 Natural History Museum, London, England, UK

**Keywords:** Cetonia aurata, Rose Chafer, genome sequence, chromosomal, Coleoptera

## Abstract

We present a genome assembly from an individual male *Cetonia aurata* (the Rose Chafer; Arthropoda; Insecta; Coleoptera; Scarabaeidae). The genome sequence is 479.6 megabases in span. Most of the assembly is scaffolded into 11 chromosomal pseudomolecules, including the X and Y sex chromosomes. The mitochondrial genome has also been assembled and is 20.85 kilobases in length. Gene annotation of this assembly on Ensembl identified 12,621 protein coding genes.

Species taxonomy.

Eukaryota; Metazoa; Eumetazoa; Bilateria; Protostomia; Ecdysozoa; Panarthropoda; Arthropoda; Mandibulata; Pancrustacea; Hexapoda; Insecta; Dicondylia; Pterygota; Neoptera; Endopterygota; Coleoptera; Polyphaga; Scarabaeiformia; Scarabaeoidea; Scarabaeidae; Cetoniinae; *Cetonia*; *Cetonia aurata* (Linnaeus, 1758) (NCBI: txid290679).

## Background

The Rose Chafer *Cetonia aurata* (Coleoptera: Scarabaeoidea: Scarabaeidae: Cetoniinae), is a medium-sized (12–19 mm) beetle. It is typically iridescent green, but can occur in a variety of colours: darker, variegated or golden, with variable white marks on the elytra. There is always a V-shaped mark on the back where the wing cases meet. ( Fremlin, 2018). *C. aurata* is distributed across the Palaearctic ( GBIF Secretariat, 2023), primarily inhabiting regions of southern and central Europe as well as south-east Asia, with scattered records through southern Britain.

Distinct from the North American Rose Chafer ( *Macrodactylus subspinosus*) and the rare Noble Chafer ( *Gnorimus nobilis*), *C. aurata* can be identified through the shape of its scutellum. The underside of the beetle has a coppery colour, and its upper side is sometimes bronze, copper, violet, blue/black, or grey. This beetle serves an ecological role as a beneficial saprophagous species, consuming decaying organic matter and floral resources such as pollen, nectar, and flowers.

The larvae of *Cetonia aurata* are C-shaped and have a firm, wrinkled, and hairy body. They overwinter in decaying organic matter like compost, manure, or rotting wood. In principle, the Rose Chafer does not cause any significant damage, but in a rare mass occurrence, feeding damage to flowers may occur. The grubs feed on dead plant parts and wood mulch and are therefore, unlike many other grubs, not harmful. On the contrary, they are particularly valuable compost inhabitants, as they can digest wood components well and actively contribute to humus formation ( Fremlin, 2008; Fremlin, 2018).

In general, the life cycle of *C. aurata* takes two years, with the larval stage lasting one year. The larvae pupate in the middle of the summer; the adults overwinter and mate the following spring. The adults feed on pollen and nectar for which they have adapted mouthparts with a unique mechanism for taking in pollen ( Karolyi *et al.*, 2009). The adults reproduce only after overwintering, mating in late spring to early summer, and oviposition occurs in decaying matter.

Previous cytogenetic work on *Cetonia aurata* reported a 20,Xy male karyotype, with a very small Y chromosome. This work also localised the nucleolus organiser regions to the short arms of a single acrocentric autosome pair ( Dutrillaux *et al.*, 2007). A later comparative study placed these findings in the context of variation in nucleolus organiser region localisation across Scarabaeoidea ( Dutrillaux &Dutrillaux, 2012).

The high-quality genome assembly of the Rose Chafer was generated as part of the Darwin Tree of Life Project, a collaborative effort to sequence all named eukaryotic species in the Atlantic Archipelago of Britain and Ireland.

## Genome sequence report

**Figure 1.**
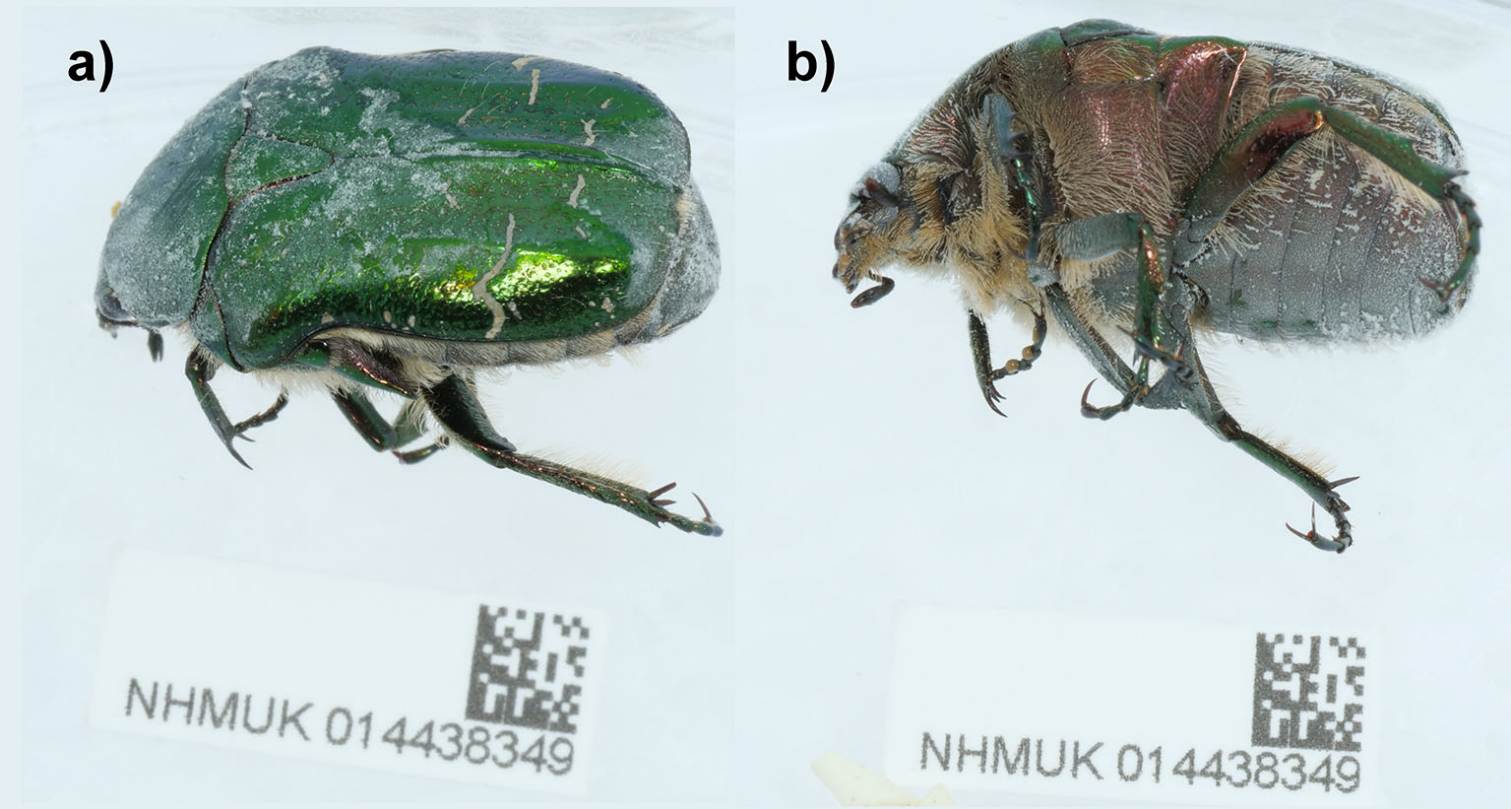
Photographs of the *Cetonia aurata* (icCetAura1) specimen used for genome sequencing.

The genome was sequenced from one male *Cetonia aurata* ( Figure 1) collected from Penhale Dunes, England (50.37, −5.14). A total of 55-fold coverage in Pacific Biosciences single-molecule HiFi long reads was generated. Primary assembly contigs were scaffolded with chromosome conformation Hi-C data. Manual assembly curation corrected 13 missing joins or mis-joins, reducing the scaffold number by 5.04%.

**Figure 2.**
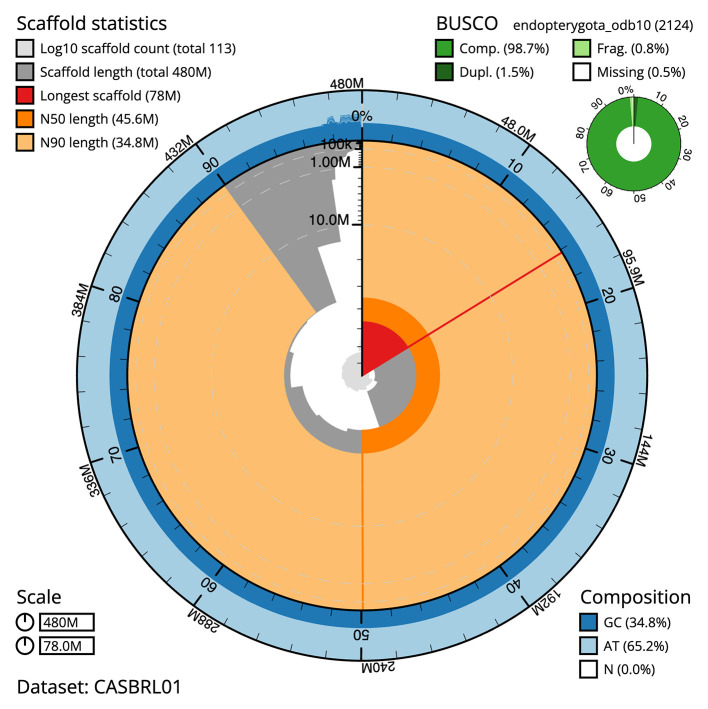
Genome assembly of *Cetonia aurata*, icCetAura1.1: metrics. The BlobToolKit Snailplot shows N50 metrics and BUSCO gene completeness. The main plot is divided into 1,000 size-ordered bins around the circumference with each bin representing 0.1% of the 479,646,749 bp assembly. The distribution of scaffold lengths is shown in dark grey with the plot radius scaled to the longest scaffold present in the assembly (77,962,046 bp, shown in red). Orange and pale-orange arcs show the N50 and N90 scaffold lengths (45,601,692 and 34,767,614 bp), respectively. The pale grey spiral shows the cumulative scaffold count on a log scale with white scale lines showing successive orders of magnitude. The blue and pale-blue area around the outside of the plot shows the distribution of GC, AT and N percentages in the same bins as the inner plot. A summary of complete, fragmented, duplicated and missing BUSCO genes in the endopterygota_odb10 set is shown in the top right. An interactive version of this figure is available at https://blobtoolkit.genomehubs.org/view/Cetonia%20aurata/dataset/CASBRL01/snail.

**Figure 3.**
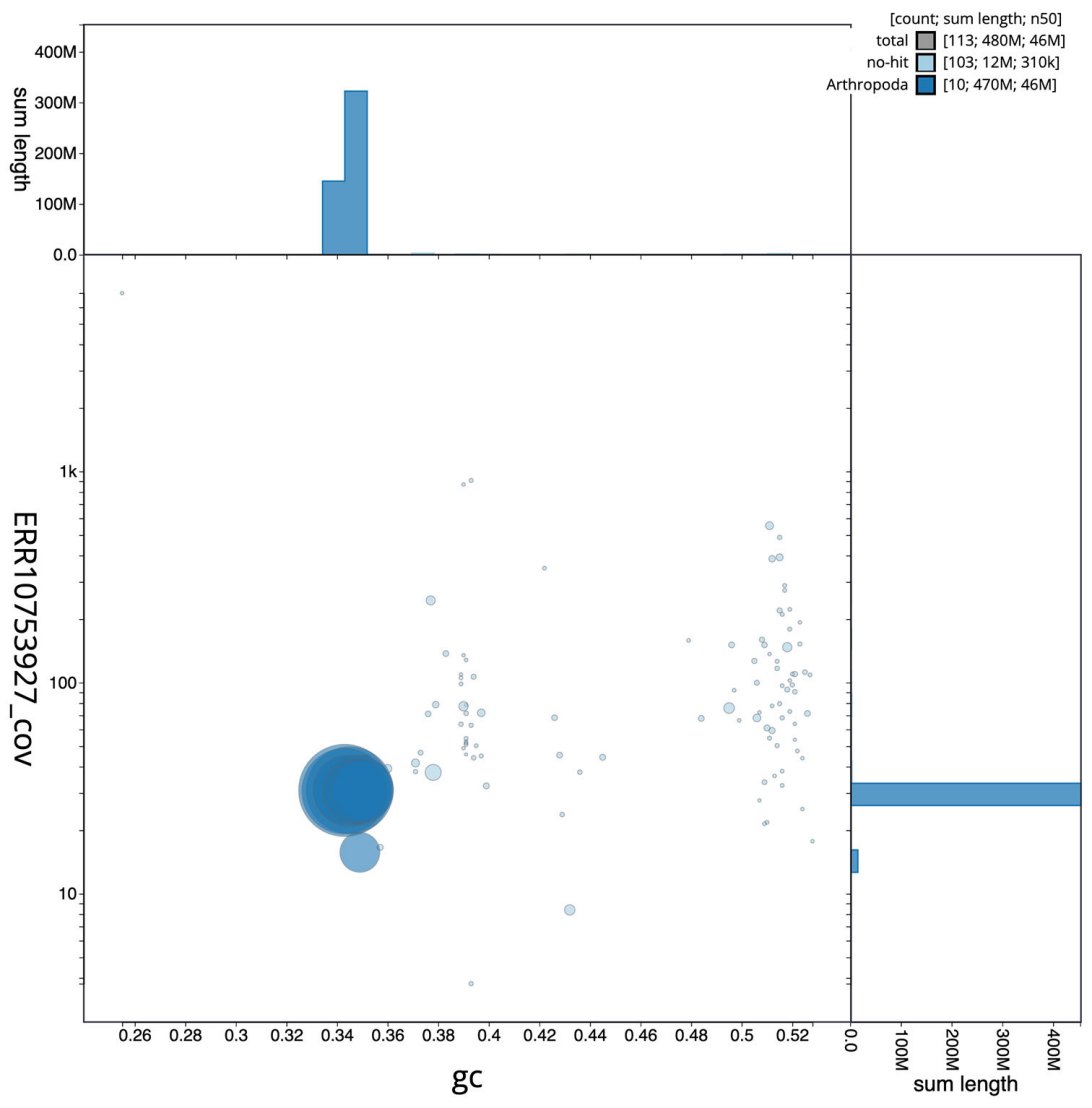
Genome assembly of *Cetonia aurata*, icCetAura1.1: BlobToolKit GC-coverage plot. Scaffolds are coloured by phylum. Circles are sized in proportion to scaffold length. Histograms show the distribution of scaffold length sum along each axis. An interactive version of this figure is available at https://blobtoolkit.genomehubs.org/view/Cetonia%20aurata/dataset/CASBRL01/blob.

**Figure 4.**
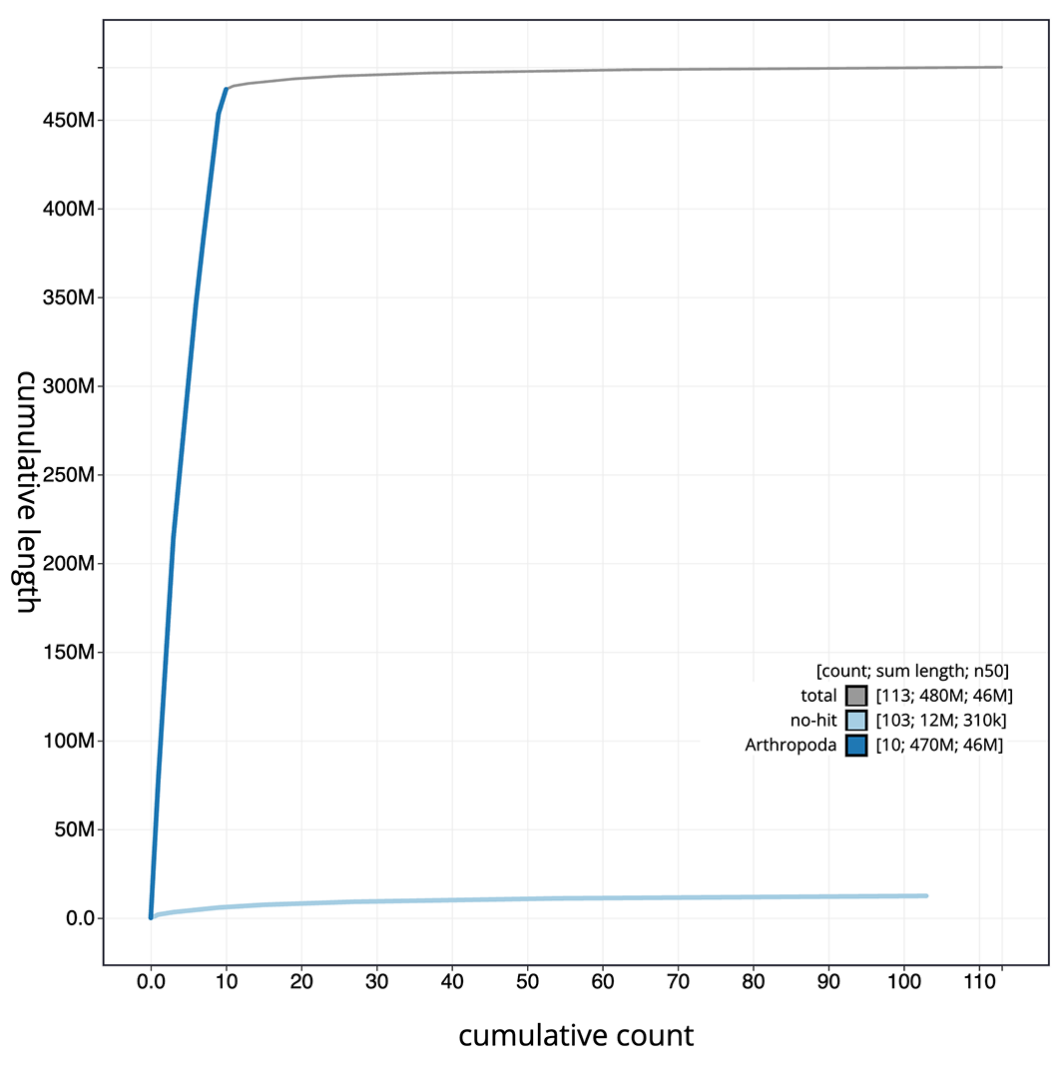
Genome assembly of *Cetonia aurata*, icCetAura1.1: BlobToolKit cumulative sequence plot. The grey line shows cumulative length for all scaffolds. Coloured lines show cumulative lengths of scaffolds assigned to each phylum using the buscogenes taxrule. An interactive version of this figure is available at https://blobtoolkit.genomehubs.org/view/Cetonia%20aurata/dataset/CASBRL01/cumulative.

**Figure 5.**
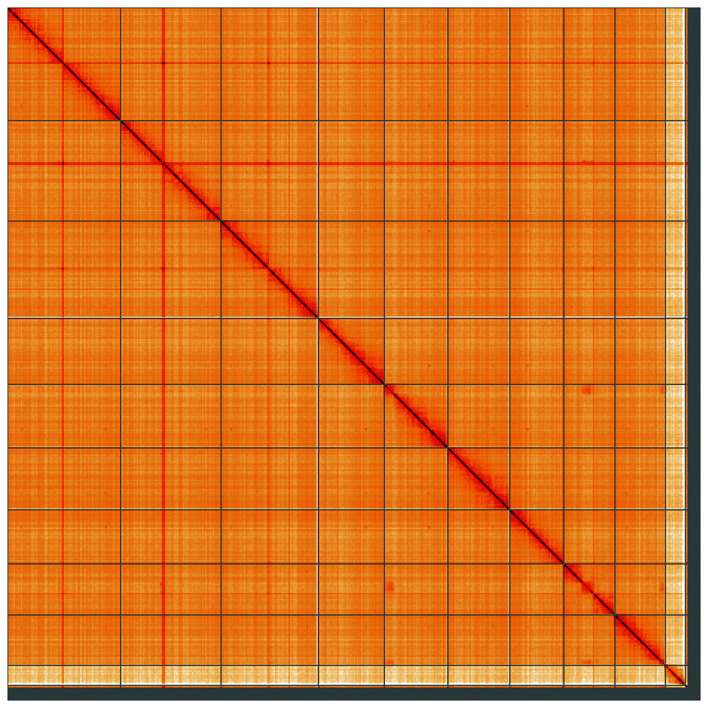
Genome assembly of *Cetonia aurata*, icCetAura1.1: Hi-C contact map of the icCetAura1.1 assembly, visualised using HiGlass. Chromosomes are shown in order of size from left to right and top to bottom. An interactive version of this figure may be viewed at https://genome-note-higlass.tol.sanger.ac.uk/l/?d=BQCKDZNBTzyjbNjt2KkpLg.

The final assembly has a total length of 479.6 Mb in 112 sequence scaffolds with a scaffold N50 of 45.6 Mb ( Table 1). The snail plot in Figure 2 provides a summary of the assembly statistics, while the distribution of assembly scaffolds on GC proportion and coverage is shown in Figure 3. The cumulative assembly plot in Figure 4 shows curves for subsets of scaffolds assigned to different phyla. Most (98.5%) of the assembly sequence was assigned to 11 chromosomal-level scaffolds, representing 9 autosomes and the X and Y sex chromosomes. Chromosome-scale scaffolds confirmed by the Hi-C data are named in order of size ( Figure 5; Table 2). While not fully phased, the assembly deposited is of one haplotype. Contigs corresponding to the second haplotype have also been deposited. The mitochondrial genome was also assembled and can be found as a contig within the multifasta file of the genome submission.

**Table 1.** Genome data for *Cetonia aurata*, icCetAura1.1.

Project accession data		
Assembly identifier	icCetAura1.1	
Assembly release date	2023-04-08	
Species	*Cetonia aurata*	
Specimen	icCetAura1	
NCBI taxonomy ID	290679	
BioProject	PRJEB58657	
BioSample ID	SAMEA11025137	
Isolate information	icCetAura1, male (DNA, Hi-C) icCetAura2 (RNA sequencing)	
Assembly metrics *		***Benchmark***
Consensus quality (QV)	Primary: 67.9; alternate: 67.5; combined: 67.6	*≥ 40*
*k*-mer completeness	Primary: 79.14%; alternate: 67.53%; combined: 99.40%	*≥ 95%*
BUSCO **	C:98.7%[S:97.2%,D:1.5%],F:0.8%,M:0.5%,n:2,124	*S > 90%; D < 5%*
Percentage of assembly mapped to chromosomes	98.5%	*≥ 90%*
Sex chromosomes	X and Y chromosomes	*localised homologous pairs*
Organelles	Mitochondrial genome assembled	*complete single alleles*
**Raw data accessions**		
PacificBiosciences SEQUEL II	ERR10753927	
Hi-C Illumina	ERR10742409	
PolyA RNA-Seq Illumina	ERR11641117	
**Genome assembly**		
Assembly accession	GCA_949128085.1	
*Accession of alternate haplotype*	GCA_949128095.1	
Span (Mb)	479.6	
Number of contigs	195	
Contig N50 length (Mb)	8.1	
Number of scaffolds	112	
Scaffold N50 length (Mb)	45.6	
Longest scaffold (Mb)	78.0	
**Genome annotation**		
Number of protein-coding genes	12,621	
Number of non-coding genes	2,411	
Number of gene transcripts	20,615	

**Table 2.** Chromosomal pseudomolecules in the genome assembly of *Cetonia aurata*, icCetAura1.

INSDC accession	Chromosome	Length (Mb)	GC%
OX421882.1	1	77.96	34.5
OX421883.1	2	69.22	34.5
OX421884.1	3	66.96	34.5
OX421885.1	4	45.6	34.5
OX421886.1	5	43.8	35.0
OX421887.1	6	42.6	34.5
OX421888.1	7	37.3	34.5
OX421889.1	8	35.24	35.0
OX421890.1	9	34.77	35.0
OX421891.1	X	13.79	35.0
OX421892.1	Y	1.87	38.0
OX421893.1	MT	0.02	25.5

[i] * Assembly metric benchmarks are adapted from column VGP-2020 of “ Table 1: Proposed standards and metrics for defining genome assembly quality” from ( Rhie *et al.*, 2021). ** BUSCO scores based on the endopterygota_odb10 BUSCO set using v5.3.2. C = complete [S = single copy, D = duplicated], F = fragmented, M = missing, n = number of orthologues in comparison. A full set of BUSCO scores is available at https://blobtoolkit.genomehubs.org/view/Cetonia%20aurata/dataset/CASBRL01/busco.

The combined primary and alternate assemblies achieve an estimated QV of 67.6. The *k*-mer completeness is 79.14% for the primary assembly, 67.53% for the alternate haplotype, and 99.40% for the combined assemblies. The primary assembly has a BUSCO v5.3.2 completeness of 98.7% (single = 97.2%, duplicated = 1.5%), using the endopterygota_odb10 reference set ( *n* = 2,124).

Metadata for specimens, barcode results, spectra estimates, sequencing runs, contaminants and pre-curation assembly statistics are given at https://links.tol.sanger.ac.uk/species/290679.

## Genome annotation report

The *Cetonia aurata* genome assembly (GCA_949128085.1) was annotated using the Ensembl rapid annotation pipeline ( Table 1; https://rapid.ensembl.org/Cetonia_aurata_GCA_949128085.1/Info/Index). The resulting annotation includes 20,615 transcribed mRNAs from 12,621 protein-coding and 2,411 non-coding genes.

## Methods

### Sample acquisition and nucleic acid extraction

A male *Cetonia aurata* (specimen ID NHMUK014438349, ToLID icCetAura1) was collected from Penhale Dunes, England, UK (latitude 50.37, longitude −5.14) on 2021-06-30 using an aerial net. The specimen was collected and identified by Andrew Grayson (Dipterists Forum) and preserved by dry freezing at −80 °C. The specimen used for RNA sequencing (specimen ID NHMUK014436794, ToLID icCetAura2) was collected from Wormwood Scrubs Park, England (latitude 51.52, longitude −0.25) on 2021-07-17. The specimen was collected and identified by Michael Geiser (Natural History Museum) and preserved on dry ice.

High molecular weight (HMW) DNA was extracted at the Tree of Life laboratory, Wellcome Sanger Institute (WSI), following a sequence of core procedures: sample preparation; sample homogenisation; HMW DNA extraction; DNA fragmentation; and fragmented DNA clean-up. The icCetAura1 sample was weighed and dissected on dry ice with tissue set aside for Hi-C sequencing (as per the protocol at https://dx.doi.org/10.17504/protocols.io.x54v9prmqg3e/v1). For sample homogenisation, thorax tissue was cryogenically disrupted using the Sample Homogenisation: Covaris cryoPREP® Automated Dry Pulverizer protocol ( https://dx.doi.org/10.17504/protocols.io.eq2lyjp5qlx9/v1). HMW DNA was extracted by means of the Automated MagAttract protocol ( https://dx.doi.org/10.17504/protocols.io.kxygx3y4dg8j/v1). HMW DNA was sheared into an average fragment size of 12–20 kb in a Megaruptor 3 system with speed setting 30, following the HMW DNA Fragmentation: Diagenode Megaruptor®3 for PacBio HiFi protocol ( https://dx.doi.org/10.17504/protocols.io.8epv5x2zjg1b/v1). Sheared DNA was purified following either the Manual solid-phase reversible immobilisation (SPRI) protocol ( https://dx.doi.org/10.17504/protocols.io.kxygx3y1dg8j/v1) for higher throughput. In brief, the method employs a 1.8X ratio of AMPure PB beads to sample to eliminate shorter fragments and concentrate the DNA. The concentration of the sheared and purified DNA was assessed using a Nanodrop spectrophotometer and Qubit Fluorometer and Qubit dsDNA High Sensitivity Assay kit. Fragment size distribution was evaluated by running the sample on the FemtoPulse system.

RNA was extracted from abdomen tissue of icCetAura2 in the Tree of Life Laboratory at the WSI using the RNA Extraction: Automated MagMax™ *mir*Vana protocol ( https://dx.doi.org/10.17504/protocols.io.6qpvr36n3vmk/v1). The RNA concentration was assessed using a Nanodrop spectrophotometer and Qubit Fluorometer using the Qubit RNA Broad-Range (BR) Assay kit. Analysis of the integrity of the RNA was done using the Agilent RNA 6000 Pico Kit and Eukaryotic Total RNA assay.

Protocols developed by the Tree of Life laboratory are publicly available on protocols.io: https://dx.doi.org/10.17504/protocols.io.8epv5xxy6g1b/v1.

### Sequencing

Pacific Biosciences HiFi circular consensus DNA sequencing libraries were constructed according to the manufacturers’ instructions. Poly(A) RNA-Seq libraries were constructed using the NEB Ultra II RNA Library Prep kit. DNA and RNA sequencing was performed by the Scientific Operations core at the WSI on Pacific Biosciences SEQUEL II (HiFi) and Illumina NovaSeq 6000 (RNA-Seq) instruments. Hi-C data were also generated from thorax tissue of icCetAura1 using the Arima2 kit and sequenced on the Illumina NovaSeq 6000 instrument.

### Genome assembly, curation and evaluation

Assembly was carried out with Hifiasm ( Cheng *et al.*, 2021) and haplotypic duplication was identified and removed with purge_dups ( Guan *et al.*, 2020). The assembly was then scaffolded with Hi-C data ( Rao *et al.*, 2014) using YaHS ( Zhou *et al.*, 2023). The assembly was checked for contamination and corrected using the gEVAL system ( Chow *et al.*, 2016) as described previously ( Howe *et al.*, 2021). Manual curation was performed using gEVAL, HiGlass ( Kerpedjiev *et al.*, 2018) and Pretext ( Harry, 2022). The mitochondrial genome was assembled using MitoHiFi ( Uliano-Silva *et al.*, 2023), which runs MitoFinder ( Allio *et al.*, 2020) and uses these annotations to select the final mitochondrial contig and to ensure the general quality of the sequence.

A Hi-C map for the final assembly was produced using bwa-mem2 ( Vasimuddin *et al.*, 2019) in the Cooler file format (Abdennur & Mirny, 2020). To assess the assembly metrics, the *k*-mer completeness and QV consensus quality values were calculated in MerquryFK ( Rhie *et al.*, 2020). The genome was analysed within the BlobToolKit environment ( Challis *et al.*, 2020) and BUSCO scores ( Manni *et al.*, 2021; Simão *et al.*, 2015) were calculated.

Table 3 contains a list of relevant software tool versions and sources.

**Table 3.** Software tools: versions and sources.

Software tool	Version	Source
BlobToolKit	4.1.5	https://github.com/blobtoolkit/blobtoolkit
BUSCO	5.3.2	https://gitlab.com/ezlab/busco
Hifiasm	0.16.1-r375	https://github.com/chhylp123/hifiasm
HiGlass	1.11.6	https://github.com/higlass/higlass
MerquryFK	1.1.2	https://github.com/thegenemyers/MERQURY.FK
MitoHiFi	2	https://github.com/marcelauliano/MitoHiFi
PretextView	0.2	https://github.com/wtsi-hpag/PretextView
purge_dups	1.2.3	https://github.com/dfguan/purge_dups
YaHS	1.2a	https://github.com/c-zhou/yahs

### Genome annotation

The Ensembl gene annotation system ( Aken *et al.*, 2016) was used to generate annotation for the *Cetonia aurata* assembly (GCA_949128085.1). Annotation was created primarily through alignment of transcriptomic data to the genome, with gap filling via protein-to-genome alignments of a select set of proteins from UniProt ( UniProt Consortium, 2019).

### Wellcome Sanger Institute – Legal and Governance

The materials that have contributed to this genome note have been supplied by a Darwin Tree of Life Partner. The submission of materials by a Darwin Tree of Life Partner is subject to the **‘Darwin Tree of Life Project Sampling Code of Practice’**, which can be found in full on the Darwin Tree of Life website here. By agreeing with and signing up to the Sampling Code of Practice, the Darwin Tree of Life Partner agrees they will meet the legal and ethical requirements and standards set out within this document in respect of all samples acquired for, and supplied to, the Darwin Tree of Life Project.

Further, the Wellcome Sanger Institute employs a process whereby due diligence is carried out proportionate to the nature of the materials themselves, and the circumstances under which they have been/are to be collected and provided for use. The purpose of this is to address and mitigate any potential legal and/or ethical implications of receipt and use of the materials as part of the research project, and to ensure that in doing so we align with best practice wherever possible. The overarching areas of consideration are:

• Ethical review of provenance and sourcing of the material.

• Legality of collection, transfer and use (national and international).

Each transfer of samples is further undertaken according to a Research Collaboration Agreement or Material Transfer Agreement entered into by the Darwin Tree of Life Partner, Genome Research Limited (operating as the Wellcome Sanger Institute), and in some circumstances other Darwin Tree of Life collaborators.

## Data availability

European Nucleotide Archive: *Cetonia aurata.* Accession number PRJEB58657; https://identifiers.org/ena.embl/PRJEB58657 ( Wellcome Sanger Institute, 2023). The genome sequence is released openly for reuse. The *Cetonia aurata* genome sequencing initiative is part of the Darwin Tree of Life (DToL) project. All raw sequence data and the assembly have been deposited in INSDC databases. Raw data and assembly accession identifiers are reported in Table 1.

## Author information

Members of the Natural History Museum Genome Acquisition Lab are listed here: https://doi.org/10.5281/zenodo.7139035.

Members of the Darwin Tree of Life Barcoding collective are listed here: https://doi.org/10.5281/zenodo.4893703.

Members of the Wellcome Sanger Institute Tree of Life programme are listed here: https://doi.org/10.5281/zenodo.4783585.

Members of Wellcome Sanger Institute Scientific Operations: Sequencing Operations collective are listed here: https://doi.org/10.5281/zenodo.10043364.

Members of the Tree of Life Core Informatics collective are listed here: https://doi.org/10.5281/zenodo.5013541.

Members of the Darwin Tree of Life Consortium are listed here: https://doi.org/10.5281/zenodo.4783558.
