# Supplementary material for: The genome sequence of the Rose Chafer, Cetonia aurata (Linnaeus, 1758)
Source: Wellcome Open Res. 2026 May 5;8:561. Originally published 2023 Dec 5. [Version 2] doi: 10.12688/wellcomeopenres.20412.2 (PMC13157610; doi:10.12688/wellcomeopenres.20412.2)
Supplement: Peer review history

# Reviewer report: Reviewer response for version 2

**DOI:** 10.21956/wellcomeopenres.29295.r154957

**Published:** 2026-05-09

**Authors:** Maria Antonia Madrid Restrepo

**Affiliations:** 1 KU Leuven, Leuven, Belgium

This data note presents a chromosome-level genome assembly for the rose chafer, *Cetonia aurata*, generated from a male specimen as part of the Darwin Tree of Life project. The final assembly spans 479.6 Mb, with 98.5% assigned to 11 chromosomal pseudomolecules, including the X and Y chromosomes, and an additional assembled mitochondrial genome. The assembly is based on PacBio HiFi data scaffolded with Hi-C, followed by manual curation, and is accompanied by an Ensembl rapid annotation reporting 12,621 protein-coding genes. Overall, this is a strong and useful reference genome resource, and the manuscript succeeds as a genome data note. The rationale for generating the dataset is clearly stated. The manuscript gives a concise biological background on *C. aurata*, including its ecology, distribution, life history, and prior cytogenetic work, and then places the sequencing effort in the context of the Darwin Tree of Life objective to generate reference genomes for named eukaryotic species from Britain and Ireland. For a data note, this level of motivation is entirely appropriate. 

 The protocols are appropriate and the work is technically sound. The methods are also sufficiently described for replication in the context of this article type. The datasets are clearly presented and accessible. 

 I have only a few minor comments and suggestions.

 First, the assembly methods are solid, but the text could be marginally improved by adding one short sentence clarifying how sex chromosomes were assigned. The manuscript reports that the X and Y chromosomes were identified and that a male karyotype of 20,Xy was previously known, but it does not explicitly explain whether X/Y assignment in the assembly was based on Hi-C structure alone, homology, coverage patterns, cytogenetic expectations, or a combination of these. This is not a flaw, but a brief clarification would make the interpretation stronger. 

 Second, although the methods are fully adequate for this format, the annotation section is brief. Since version 2 now includes the annotation results in the abstract and body, it would be helpful to include one extra quality statement for the annotation, for example annotation BUSCO or a brief summary of transcript/protein support, if available from the Ensembl rapid pipeline. Third, because the primary assembly k-mer completeness is reported as 79.14%, whereas the combined primary and alternate assemblies reach 99.40%, one sentence of interpretation would help readers unfamiliar with partially phased assemblies. 

 Overall, this is a well-executed genome data note describing a high-quality chromosomal assembly for *Cetonia aurata*. The manuscript is appropriately focused, the assembly methods are current and sound, and the resulting genomic resource should be valuable for comparative and evolutionary studies in beetles and related insects.

Are sufficient details of methods and materials provided to allow replication by others?

Yes

Is the rationale for creating the dataset(s) clearly described?

Yes

Are the datasets clearly presented in a useable and accessible format?

Yes

Are the protocols appropriate and is the work technically sound?

Yes

Reviewer Expertise:

Bioinformatics, genome assembly, population genetics

I confirm that I have read this submission and believe that I have an appropriate level of expertise to confirm that it is of an acceptable scientific standard.

# Reviewer report: Reviewer response for version 1

**DOI:** 10.21956/wellcomeopenres.22600.r101972

**Published:** 2024-10-08

**Authors:** Sean D Schoville

**Affiliations:** 1 Department of Entomology, University of Wisconsin-Madison, Madison, Wisconsin, USA

The paper presents the first genome assembly of the scarab beetle, Cetonia aurata. The combined HiFi sequence data and HiC data have been used to generate a chromosomal-level assembly, including both X and Y sex chromosomes. This will undoubtedly provide a useful resource for scientists studying comparative genomics of insects, especially beetles, as the number of genomes remains limited. The BUSCO annotation and other supporting analyses suggest it is a robust assembly.

 I think it would be helpful to provide an annotation report for the gene set, perhaps using BUSCO completeness scores.

**Minor typographical revisions:**

 Its general practice to abbreviate genus names after first usage, except when it starts a new sentence. In the first paragraph of the Background, you start a sentence with "C. aurata" and this should be changed to Cetonia aurata". In the third paragraph, you spell out the name, although it is in the middle of a sentence and should at this point be abbreviated as "C. aurata." Please examine your usage of latin names throughout.

 There is an incomplete sentence at the end of the first paragraph of the methods "preserved on...".

 I wonder if you could try to determine the sex of the specimen used to make the RNA sample, simply by using coverage on the X and Y chromosomes. This is missing from the metadata.

 In the paragraph on Sequencing, there is a tagged item that needs to be replaces "$HIC_TISSUE".

Are sufficient details of methods and materials provided to allow replication by others?

Yes

Is the rationale for creating the dataset(s) clearly described?

Yes

Are the datasets clearly presented in a useable and accessible format?

Yes

Are the protocols appropriate and is the work technically sound?

Yes

Reviewer Expertise:

Evolutionary genomics of insects

I confirm that I have read this submission and believe that I have an appropriate level of expertise to confirm that it is of an acceptable scientific standard.

# Reviewer report: Reviewer response for version 1

**DOI:** 10.21956/wellcomeopenres.22600.r73994

**Published:** 2024-05-28

**Authors:** Ayoob Obaid Alfalahi

**Affiliations:** 1 University of Anbar, Ramadi, Al Anbar Governorate, Iraq

I found the article is really interesting through which the genome sequence of the Rose Chafer, *Cetonia aurata* will participate effectively in generating high quality data, and I think it will be particularly valuable for future research. The mitochondrial genome of *C. aurata* was assembled too. However, after thoroughly reading the manuscript, I'm sending the following comments:

1. In the background section, authors still able to include some up-to-date relevant references, including these important references may be necessary for supporting these claims especially the morphological description.
2. Images of different insect development stages are crucial for accurate morphological characterization; especially in such important genomic studies.
3. Image 1 was actually two images referred to as (a and (b. However, the image caption should clarify the difference between the two images.   
4. Methodology section is informative and satisfactorily detailed.
5. I think there is something missing on page #5; you may double check the end of the paragraph in the first column.
6. The majority of the used keywords are already included in the manuscript's Title! However, using relevant descriptive Keywords other than those mentioned in the title can help increase your manuscript's visibility in article searches.

I wish authors will able to use these comments to improve their article before re-submission.

 Best regards

Are sufficient details of methods and materials provided to allow replication by others?

Yes

Is the rationale for creating the dataset(s) clearly described?

Yes

Are the datasets clearly presented in a useable and accessible format?

Yes

Are the protocols appropriate and is the work technically sound?

Yes

Reviewer Expertise:

Plant Biotechnology

I confirm that I have read this submission and believe that I have an appropriate level of expertise to confirm that it is of an acceptable scientific standard, however I have significant reservations, as outlined above.
